# Role of *Streptococcus pneumoniae* Proteins in Evasion of Complement-Mediated Immunity

**DOI:** 10.3389/fmicb.2017.00224

**Published:** 2017-02-20

**Authors:** Greiciely O. Andre, Thiago R. Converso, Walter R. Politano, Lucio F. C. Ferraz, Marcelo L. Ribeiro, Luciana C. C. Leite, Michelle Darrieux

**Affiliations:** ^1^Laboratório de Biologia Celular e Molecular de Microrganismos, Universidade São FranciscoBragança Paulista, Brazil; ^2^Centro de Biotecnologia, Instituto ButantanSão Paulo, Brazil; ^3^Programa de Pós-graduação Interunidades em Biotecnologia, Universidade de São PauloSão Paulo, Brazil; ^4^Laboratório de Farmacologia, Universidade São FranciscoBragança Paulista, Brazil

**Keywords:** complement system, *Streptococcus pneumoniae*, virulence factors pneumococcal surface proteins, pneumococcal moonlighting proteins, protein-based vaccines

## Abstract

The complement system plays a central role in immune defense against *Streptococcus pneumoniae*. In order to evade complement attack, pneumococci have evolved a number of mechanisms that limit complement mediated opsonization and subsequent phagocytosis. This review focuses on the strategies employed by pneumococci to circumvent complement mediated immunity, both *in vitro* and *in vivo*. At last, since many of the proteins involved in interactions with complement components are vaccine candidates in different stages of validation, we explore the use of these antigens alone or in combination, as potential vaccine approaches that aim at elimination or drastic reduction in the ability of this bacterium to evade complement.

## Introduction

The complement system is an important mechanism in human immunity, with more than thirty proteins produced in soluble phase by the liver or expressed in cell surfaces [reviewed in (Ritchie et al., 2002; Dunkelberger and Song, 2010; Merle et al., 2015a)]. This system comprises a set of recognition molecules present in the plasma and interstitial fluids that are quickly activated in response to pathogens such as bacteria, yeast and virus, as well as infected cells and damaged tissues (Dunkelberger and Song, 2010; Ehrnthaller et al., 2011). Complement activation is triggered by serine protease domains present in components that become active and cleave the next protein in a cascade-like manner [reviewed in (Ehrnthaller et al., 2011; Merle et al., 2015a)]. The proteolytic cleavage steps generate fragments that bind to the microbial surfaces thereby acting as opsonins, promoting a more efficient phagocytosis and releasing peptides into the bloodstream, which are able to induce inflammatory responses [reviewed in (Merle et al., 2015b)]. Complement activation also promotes assembly of the membrane attack complex (MAC), which is capable of forming pores on membranes, causing osmotic lysis (Esser, 1994; Ritchie et al., 2002).

*Streptococcus pneumoniae* is a common colonizer of the human nasopharynx and is usually asymptomatic; however, in susceptible hosts it can invade other niches, causing otitis media, conjunctivitis, pneumonia, meningitis, and septicaemia (Kadioglu et al., 2008). The immune response against pneumococcal infections is highly supported by complement activities such as opsonization and activation of inflammatory responses (Janoff et al., 1999; Jarva et al., 2003). In order to evade the anti-bacterial effects of complement, pneumococci have developed many virulence factors that impair complement activity, thus contributing to bacterial evasion from the immune system (Jarva et al., 2003).

The current prophylaxis against pneumococcal diseases is based on polysaccharide vaccines (alone or conjugated to protein carriers), which have proven to be effective against invasive disease (Braido et al., 2008). However, high production costs and a reduced number of polysaccharide serotypes included in the formulations (a limitation of the conjugation process) have hampered the implementation of these vaccines in lower income countries, which are greatly affected by pneumococcal diseases (Croney et al., 2013). This scenario encourages the efforts to find alternative vaccines that can offer higher coverage at a reduced cost, as well as increasing protection against non-invasive disease (Moffitt and Malley, 2011; Darrieux et al., 2015). Based on these considerations and the role of bacterial proteins in complement attack, many proteins have been evaluated as vaccines in animal infection models, with encouraging outcomes. The employment of proteins that have roles in the inhibition of complement as vaccine antigens could promote the blockage of anti-complement abilities of pneumococci and result in more effective opsonophagocytosis. In addition, the combination of proteins may trigger broader, more effective immune responses compared to the use of one antigen alone. Therefore, the present review discusses the role of pneumococcal proteins in complement evasion, as well as the potential of using combinations of these proteins as pneumococcal vaccines.

## Contribution of the Complement System to Host Immunity Against Pneumococci

**FIGURE 1:**
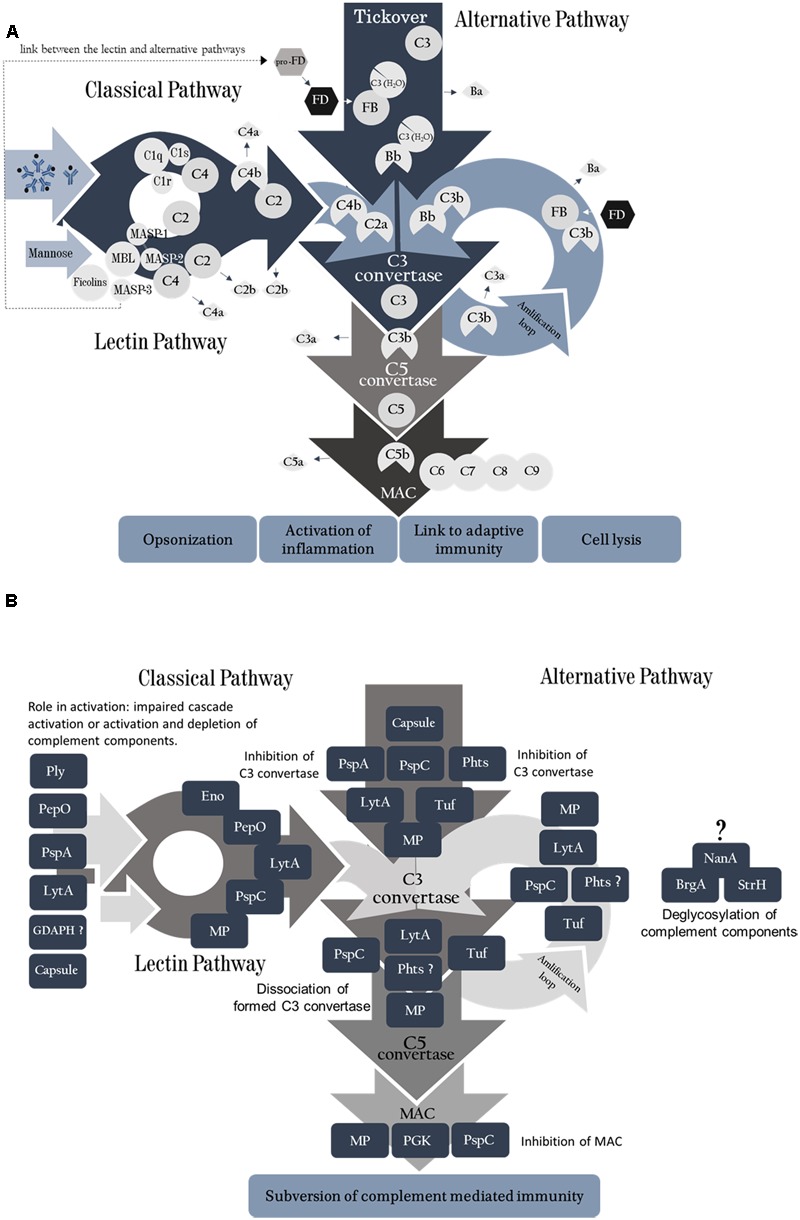
**(A)** Complement System activation pathways. The classical pathway is activated by binding of C1q molecules to IgM or IgG. C1s cleaves C4 into C4a and C4b, further degrading C2 when bound with C4b, forming C2b (free molecule) and C2a, which remains bound to C4b forming C4bC2a (C3 convertase of the classical pathway). The lectin pathway is activated in a similar fashion, by binding of MBL molecule to mannose or other sugars on microbial surfaces. It can also be initiated by ficolins or collectins. Upon target binding, MASP-1 autoactivates first, and then it activates MASP-2. MASP-2 then cleaves C4 and both enzymes cleave C2, generating the classical C3 convertase (C4b2a). A third serine protease, MASP-3, is able to activate pro-Factor D (pro-FD) in the resting human blood, favoring alternative complement pathway activation. The Alternative pathway is activated continuously by spontaneous hydrolysis of C3 into C3 (H_2_O); FB binds to this molecule and is split up by FD, yielding Ba and Bb that remains bound to form C3 (H_2_O)Bb (the initial alternative pathway C3 convertase). This convertase is able to cleave C3 in the blood, generating C3a and C3b, which attaches to the activating surface and recruits FB. Binding of C3b with the cleavage product of FB, Bb, generates the second C3convertase of the alternative pathway, C3bBb. The amplification loop is initiated when plasmatic C3 is cleaved by C3 convertase from all three pathways yielding more C3b, which may lead to more convertase or promote microbial opsonization. The C5 convertase is formed by association of one C3b molecule to the C3 convertase (in all pathways). This aggregate cleaves C5 molecules into C5a (anaphylatoxin) and C5b, which is the platform for C6 binding. Following the cascade, C7, C8, and C9 (16–18 units) molecules bind to the activated surface to form the membrane attack complex. **(B)** Pneumococcal virulence factors act on different points of the complement cascades. Each pneumococcal antigen is depicted in relation to the complement components they interact with. PLY, GDAPH and PepO promote activation and depletion of complement components. PspA inhibits activation of both classical (CP) and alternative pathways (AP) C3 convertases; LytA inhibits classical pathway activation. PspC, PepO, Eno, and LytA undermine CP C3 convertase formation. PspC, Phts, LytA, and Tuf impair AP C3 convertase formation, as well as the amplification loop. PGK and PspC inhibit MAC assembly. NanA, BgaA, and StrH remove sialic acids from complement components, limiting complement mediated phagocytosis; they inhibit AP, but the mechanism responsible for the inhibition is not clear. The pneumococcal moonlighting proteins (MP) have indirect influence on complement through their interaction with PLG and consequent degradation of complement components.

As a classic extracellular bacterium, antibody-enhanced complement-mediated phagocytosis is an essential mechanism of pneumococcal clearance from the host (Brown et al., 1983; Paterson and Orihuela, 2010). Infection with *S. pneumoniae* activates all three pathways of the Complement System (**Figure 1A**), which converge in the formation of C3b and generate other molecules involved in opsonophagocytosis and activation of inflammatory responses (Supplementary Table 1).

The classical pathway is activated by specific antibodies and by recognition of cell wall phosphorylcholine by natural IgM (Brown et al., 2002). In the absence of antibodies, SIGN-R1, a transmembrane C-type lectin expressed by macrophages, recognizes carbohydrates on the pneumococcal surface and binds to C1q (Kang et al., 2006). Moreover, polymeric IgA molecules can activate complement on the pneumococcal surface (Janoff et al., 1999). More recently, it has been demonstrated that C1q acts as a bridge between pneumococci and host tissues *in vitro*. C1q bound to pneumococcal surface proteins through its C-terminal globular head region, in an antibody-independent fashion. Furthermore, C1q bound to pneumococci was able to interact with respiratory cells (Agarwal et al., 2013a) – possibly through glycosaminoglycan receptors (GAG) on their surface (Almeda et al., 1983) – promoting bacterial adherence and invasion (Agarwal et al., 2013a). The consequences of this interaction have not been investigated *in vivo*, although C1q bound simultaneously to pneumococci and host cells is still able to promote complement activation on pneumococcal cells (Agarwal et al., 2013a).

After recognition, the C1 complex becomes active and cleaves C4 into C4a – released to the soluble phase – and C4b, which remains attached to the bacterial surface; C2 binds to C4b and is split up by C1s yielding C2a – that remains bound to C4b – and C2b. The C4b2a, termed C3 convertase of the classical pathway, is able to cleave plasmatic C3 into C3a (anaphylatoxin) and C3b. C3b plays a pivotal role in the complement system; it is required to form the C5 convertase complex, when additional C3b binds to C3 convertase [reviewed in (Ehrnthaller et al., 2011; Merle et al., 2015a)]. Formation of C5 convertase marks the late stage of complement activation, which culminates with the assembly of MAC [reviewed in (Esser, 1994; Ehrnthaller et al., 2011)]. As a Gram-positive pathogen, the thick peptidoglycan layer on the pneumococcal cell wall limits the effects of MAC attack (Jarva et al., 2003); yet, the complement opsonic and pro-inflammatory functions are essential for the control of pneumococcal infections (Brown et al., 1983; Paterson and Mitchell, 2006), facilitating bacterial phagocytosis and clearance. In that matter, C3b attached to microbial surfaces may act as an opsonin, or generate the amplification loop, where C3b associates with Bb, a protein of alternative pathway, promoting more C3 convertase formation [reviewed in (Ehrnthaller et al., 2011; Merle et al., 2015a)].

The importance of the classical pathway for immunity against pneumococci is further supported by the observation that deficiency in classical complement components such as C1q, C4 and C2 is associated with recurrent and severe pneumococcal infections (Rupprecht et al., 2007; Yuste et al., 2008). Similarly, mouse models of complement deficiency (either by genetic manipulation or transient depletion) reveal an increased susceptibility to pneumococcal sepsis (Brown et al., 2002).

The lectin pathway is initiated when Mannose-binding lectin (MBL), collectin-10, collectin-11, or ficolins-1, 2, or 3 bind to target surfaces and interact with the serine proteases MASP-1, 2, and 3. Upon target binding, MASP-1 can self-activate and is required for activation of MASP-2. MASP-2 then cleaves C4 and both enzymes cleave C2, generating the classical C3 convertase (C4b2a). A third serine protease, MASP-3, is able to activate pro-Factor D [pro-FD) in the resting human blood, favoring alternative complement pathway activation [reviewed in (Dobo et al., 2016)]. The lectin pathway plays a less crucial but still important role in complement-mediated immunity against pneumococci (Ali et al., 2012; Garcia-Laorden et al., 2013). While a previous report found no clear role for MBL in pneumococcal infections (Brown et al., 2002), more recent studies found an important contribution for the lectin pathway in mice that do not express ficolins (Endo et al., 2012) or MASP-2 (Ali et al., 2012), which showed an increased susceptibility to pneumococcal infections and a reduced opsonizing capacity in non-immune hosts (Ali et al., 2012; Endo et al., 2012). Different components on the pneumococcal surface interact with lectins: L-ficolin binds to the capsular polysaccharides 11A, 11D, and 11F, and to phosphorylcholine residues (ChoP) on the bacterial cell wall, while M-ficolin interacts with N-acetylmannosamine in capsular types 19B and 19C (Kjaer et al., 2013). As a result of this recognition, the complement proteolytic cascade continues as described for the classical pathway [reviewed in (Merle et al., 2015a)].

As observed for the classical pathway, genetic deficiency in MBL is related to an increased susceptibility to pneumococcal disease (Roy et al., 2002). A meta-analysis on human studies suggested that MBL deficiency may be associated with susceptibility to invasive pneumococcal disease (Garcia-Laorden et al., 2013). This was confirmed by Brouwer et al. (2013) in a cohort study, showing that MBL deficiency was associated with a considerable increase in susceptibility to meningitis caused by *S. pneumoniae* (Brouwer et al., 2013).

The alternative pathway is initiated when C3 (H_2_O) – resulting from spontaneous hydrolysis of C3 (Pangburn et al., 1981) – is recognized by factor B (FB) which is then cleaved in factor Ba and factor Bb by factor D (FD); Bb remains attached to C3 (H_2_O), forming the soluble phase C3 convertase of the alternative pathway. This convertase is able to cleave C3 in the blood, generating C3a and C3b; if C3b is formed close enough to the cell membrane, it will covalently attach to that surface and, because it is structurally similar to C3 (H_2_O), it will recruit FB and the cascade will proceed on the activating surface (Pangburn et al., 1983). The activation cascade triggered by the alternative pathway was found to be related to the density of C3b deposited on the bacterial surface (Jarva et al., 2003), which can be translated in more opsonization of pneumococci. Also, the observation that knockout mice for alternative pathway proteins, such as factor B and factor D, are more susceptible pneumococcal infections by strains which lack important virulence factors strongly suggests that this complement pathway contributes to pneumococcal clearance by the host. This will be discussed in more detail further.

Other molecules – including C-reactive protein (CRP) (Biro et al., 2007; Gang et al., 2015), surfactant protein A (S-PA) (Watford et al., 2001; Madhukaran et al., 2015), serum amyloid P (SAP) (Yuste et al., 2007) and molecules of the coagulation cascade (Muhlfelder et al., 1979; Sims et al., 1988; Amara et al., 2010; Rayes et al., 2014; Kim et al., 2015) can activate complement. Particularly CRP and SAP recognize *S. pneumoniae* and induce complement activation on this bacterium (Yuste et al., 2007; Gang et al., 2015). Both have collagen binding sites which promote binding to C1q and are able to interact with polysaccharides (PS), lipopolysaccharides (LPS), and phosphocholine (PCh) on microbial surfaces (Kaplan and Volanakis, 1974; Szalai et al., 1995; Yuste et al., 2007; Gang et al., 2015). CRP was shown to protect mice against pneumococcal infection by binding to Phosphocholine (PCh), but also by mechanisms independent on PCh (Gang et al., 2015). Interestingly, CRP has been implicated in the regulation of complement by binding to regulators, C4BP and factor H, modulating the classical and alternative pathways, respectively (Biro et al., 2007). A second role of CRP on complement activation has been shown in pneumococci lacking PspC, where the presence of CRP increased binding to FH (Jarva et al., 2002). SAP has also been shown to increase complement deposition on pneumococci by the classical pathway, leading to a more efficient bacterial clearance; mice genetically deficient in SAP were more susceptible to fatal pneumococcal infection, a condition that was partially ameliorated by supplementation with human SAP (Yuste et al., 2007).

The main result of complement activation by *S. pneumoniae* is an increase in bacterial phagocytosis. Alveolar and monocyte-derived macrophages play a central role in pneumococcal clearance during subclinical pulmonary infections, mainly through production of reactive nitrogen species (MacMicking et al., 1997). Apoptosis has also been demonstrated to aid pneumococcal clearance by macrophages which have exhausted their capacity to kill ingested bacteria, in a nitric oxide (NO) dependent manner [reviewed in (Aberdein et al., 2013)]. This initial response is often enough to eliminate the invading bacteria, with no clinical signs of disease. However, when macrophages exceed their clearance capacity and can no longer control bacterial spread, Th cells and neutrophils are recruited. Production of Th1 and Th17 CD4^+^ cytokines contributes to bacterial clearance, with activation of inflammatory responses. Neutrophils are the main phagocytes controlling pneumococcal loads at this stage; they produce proteases – including elastase and cathepsin G – which mediate effective killing of ingested bacteria [reviewed in (Aberdein et al., 2013)]. Phagocytosis of pneumococci by this cell type is highly dependent on complement activation. However, an exacerbated inflammatory response leads to tissue damage and can be deleterious to the host.

Complement-mediated phagocytosis is also an important mechanism of bacterial clearance during otitis media caused by pneumococci, preventing bacterial spread; neutrophils from the circulation reach the middle ear where they recognize and kill bacteria coated with complement products (Sabharwal et al., 2009). Also, a variation in bacterial resistance to phagocytosis in this niche has been associated with capsule production (Li et al., 2012). Finally, the role of the complement system on nasopharyngeal colonization by pneumococci is related to the prevention of sepsis following mucosal colonization, and not to the bacterial loads in the nasopharynx (Bogaert et al., 2010).

## Pneumococcal Virulence Factors Interfere with Complement Action

**FIGURE 2:**
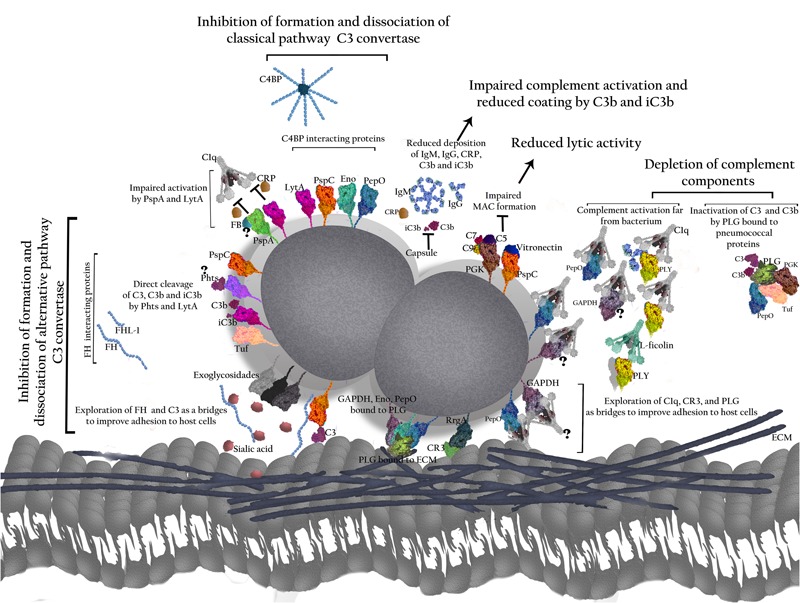
**Role of pneumococcal virulence factors in evasion from the Complement System.** The polysaccharide capsule prevents binding of IgG, IgM, and CRP to the bacterial surface, as well as C3b and iC3b, thereby impairing complement activation by classical (CP) and alternative pathways (AP). The proteins have been grouped according to their interactions with complement. T indicates direct inhibition by pneumococcal antigens. PspA affects C3 convertase formation by interfering with FB and prevents CRP binding to phophocholine on the bacterial wall, thus inhibiting activation of the classical pathway. PspC binds to FH and C4BP promoting the inhibition of C3 convertase formation and accelerating C3 convertase decay. PspC also cleaves C3 molecules generating products that cannot activate complement. Binding of PspC to Vitronectin reduces MAC formation. Phts bind to FH and cleave C3. LytA binding to FH and C4BP reduces C3 convertase formation and promotes its dissociation. It also inhibits the interaction between CRP and C1q. In addition, LytA is able to split up C3b and iC3b. PLY released from the bacterium activates the classical pathway through interactions with IgG, C1q, and L-ficolin, and depletes complement. The exoglycosidases NanA, BgaA, and StrH remove sialic acid (SA) from complement components; also, SA favors FH activity in regulation of C3 convertase. PepO binds to C1q and activates complement when released from the pneumococcal surface, leading to depletion of complement components. PepO binding to C4BP results in down regulation of classical pathway activation. Eno decreases C3 convertase formation by binding to C4BP. GAPDH on its free form or attached to the bacterial surface binds to C1q, likely promoting complement activation. PGK impairs MAC assembly by binding to C5, C7, and C9. Tuf binds to FH and FHL-1 inhibiting formation and accelerating dissociation of C3 convertase. Tuf can also bind to CFHR-1, but the implications of this interaction are not completely elucidated. The main result of such interactions is an impaired bacterial phagocytosis. Also, pneumococcal proteins use complement molecules as bridges to interact with host receptors and favor bacterial adherence and invasion. PspC exploits FH to adhere and invade host cells. PepO uses C1q to increase bacterial adherence. RrgA improves colonization by binding to complement receptor 3 (CR3). Finally, the ability to interact with PLG – as demonstrated for PepO, Eno, and GAPDH with PLG was shown to degrade the host extracellular matrix components (ECM) and to improve invasion, while PepO, PGK and Tuf bound to PLG inactivate C3 and C3b.

The ability to resist opsonization by C3 is considered crucial not only during systemic infection, but also for persistence of pneumococci in the host nasopharynx (Brown et al., 2002; Kerr et al., 2005; Paterson and Mitchell, 2006; Paterson and Orihuela, 2010) allowing further spread to other host niches. In order to evade complement attack, the pneumococcus has evolved multiple virulence factors that contribute to complement resistance. A thick polysaccharide capsule acts in conjunction with several surface proteins and toxins to limit complement activation and/or accelerate complement products decay. The importance of each of these molecules to complement evasion will be discussed in the following sections. **Table 1** and **Figures 1B** and **2** summarize the role of the individual components on complement.

**Table 1:** Pneumococcal virulence factors: anticomplement activities.

Factors	Target molecule	Effects
Capsule	IgM, IgG, CRP, C3, and iC3b	Prevents complement activation. Inhibits bacterial opsonization by C3b and iC3b.
PspA^a^	CRP and FB	Inhibits CRP deposition and C3 convertase formation
PspC	FH, C4BP, Vitronectin, C3	Inhibits C3 convertase formation and MAC assembly.
Ply	C1q, IgG, and L-Ficolin	Activates complement away from bacterial cells and depletes complement components.
Phts^a∗^	FH and C3	Inhibits C3 convertase formation.
NanA/BgaA/StrH	-	Deglycosylate complement components.
LytA^b^	FH, C4BP, C3b, and iC3b	Inhibits C3 convertase formation and reduces opsonization by iC3b.
LytB^a^	-	Lytic activity promotes bacterial dispersion which limits complement deposition
PepO^c^	C1q, C4BP, PLG	Activates and depletes complement. Inhibits C3 convertase formation.
GAPDH^acd^	C1q, PLG	Activates and depletes complement.
Eno^cd^	C4BP, PLG	Inhibits C3 convertase formation and depletes complement.
PGK^e^	C5, C7, C9, and PLG	Inhibits MAC formation and depletes complement.
Tuf_sp_^e^	FH, FHL-1, CFHR-1, and PLG	Inhibits C3 convertase formation and depletes complement.
RrgA	CR3	Promote adhesion to and invasion of macrophages.

*^a^Possible mechanism; not completely elucidated.*

*^b^Indirect activity. Inhibits binding between C1q and CRP.*

*^c^Explores PLG in order to improve adhesion and colonization.*

*^d^Possibly cleaves/depletes complement components by binding to PLG, but this was not investigated.*

*^e^Binding to PLG possibly improves adherence and invasion, but this was not investigated.*

*^∗^Two studies found conflicting results for Pht binding to FH.*

### Pneumococcal Polysaccharide Capsule: The Major Virulence Factor

The vast majority of clinically important isolates of *S. pneumoniae* are surrounded by a polysaccharide capsule with a highly diverse composition; 97 different capsular types have been identified to date (Geno et al., 2015). The capsule inhibits the activation of both classical and alternative complement pathways, protecting pneumococci against phagocytosis (Hyams et al., 2010a). The bulky polysaccharide structure limits binding of complement molecules, and also prevents the interaction of surface-bound complement fragments with receptors on host cells. Additionally, the capsule inhibits degradation of C3b bound to the pneumococcal surface into iC3b (Hyams et al., 2010a), resulting in a decreased iC3b/C3b ratio, which could impact on complement-mediated bacterial interaction with CR3 on phagocytes. Therefore, protection afforded by the capsule is able to reduce the opsonic capacity of the complement system.

It has been demonstrated that the capsule inhibits binding of IgG, IgM, and CRP to *S. pneumoniae* (Hyams et al., 2010a). In the same work, Hyams et al. (2010a) used C1q-deficient serum (unable to activate the classical pathway) to show an increase in alternative pathway-mediated C3b/iC3b deposition on non-encapsulated strains. Similar results were found in other studies, which suggest a role for capsule in the blockage of both classical and alternative complement pathways (Abeyta et al., 2003; Hyams et al., 2010a, 2013). Noteworthy, variations in the ability of different pneumococcal serotypes to interfere with PspC-mediated FH binding showed a negative correlation with their invasiveness (Hyams et al., 2013). In that work, the levels of FH binding directly impacted on the C3b/iC3b deposition and neutrophil association, suggesting that pneumococcal capsule types may influence phagocytosis through their effects on FH binding. The polysaccharide capsule also plays a role in preventing the immune adherence of pneumococci – a complement-dependent process in which the bacteria bind to erythrocytes, are transported and transferred to macrophages, which results in reduction of the phagocytic activity against encapsulated pneumococci (Li et al., 2009).

Interestingly, the extension of complement resistance provided by the capsule varies depending on the bacterial serotype. Mutant strains with a TIGR4 background and capsular types 4 or 7F were more resistant to complement deposition and phagocytosis than those expressing polysaccharides 6A or 23F (Hyams et al., 2010b). Furthermore, this increased resistance to complement deposition correlated with an increased virulence for those strains in a mouse model of pneumococcal sepsis. Accordingly, a study investigating a serotype 11A pneumococcal strain found a correlation between the low invasiveness of this isolate and the presence of O-acetylated epitopes in the polysaccharide structure, which were recognized by ficolin-2 and activated the lectin pathway, leading to bacterial phagocytosis (Brady et al., 2014).

On a whole, the evidence suggests that capsular polysaccharides play a central role in pneumococcal escape from complement system through multiple mechanisms, impacting the bacterial ability to cause disease.

### Pneumococcal Proteins as Sentinels against the Complement System

Besides the capsule, pneumococci express many proteins that interact with the complement system and promote an increased survival against this important host defense mechanism. These molecules contribute to virulence, as they are essential to the progression of disease and usually found on the bacterial surface, cytosol, in the cell wall or even being released to the environment. Among the anti-complement proteins are PspA, PspC, Phts, LytA, LytB, LytC, NanA, SrtH, BrgA, PLY, PGK, Eno, GAPDH, PepO, and Tuf, which will be discussed individually in the following sections.

#### Pneumococcal Surface Protein A (PspA)

Pneumococcal surface protein A is present in virtually all pneumococcal isolates, independently of the serotype. It attaches to phosphocholine residues on the cell wall through the C-terminus, while the N-terminal domain is exposed on the bacterial surface (Yother and Briles, 1992); this region presents a pattern of sequence variation that was used to classify PspA proteins into six clades, included in three families (Hollingshead et al., 2000). All families are involved with inhibition of complement activation (Tu et al., 1999; Ren et al., 2003, 2004b) in the early phases of infection (Ren et al., 2004b), although the molecular mechanism of PspA interaction with complement has not been completely elucidated. A recent study demonstrated that PspA competitively inhibits binding of CRP to the phosphocholine moieties on the bacterial wall, thus limiting complement deposition through the classical pathway (Mukerji et al., 2012).

The inhibition of C3 deposition by PspA was attested by the observation that pneumococci lacking PspA were less virulent and more susceptible to C3 deposition than the wild-type strain, while the virulence of the PspA^-^ strain was recovered in complement factor B-deficient knockout mice (Tu et al., 1999). This evidence suggests that the inhibitory role of PspA over complement is related to FB activation, indicating an important contribution for the alternative complement pathway. In contrast, an *in vitro* study from (Ren et al., 2004b) found that C3b deposition on mutant pneumococci lacking PspA was dependent on the classical pathway activation, since blockage of the classical pathway (with EGTA) – but not the alternative pathway (using sera from FB deficient mice) – completely abrogated C3 deposition in the absence of PspA. However, when both complement pathways were present, C3 deposition on the PspA^-^ strain was much greater than with either pathway alone (Ren et al., 2004b). The authors conclude that while the classical pathway is required for activation of complement on PspA^-^ pneumococci, the alternative pathway is responsible for the amplification of C3 deposition (Ren et al., 2004b).

A study investigating the effects of PspA on complement mediated defense against pneumococci *in vivo* demonstrated a role for complement receptors CR1/2, since bacteria lacking PspA become virulent in knockout mice for this receptor. Also, mice that did not express factor D were more susceptible to infection with the PspA^-^ strain, confirming the importance of the alternative pathway for PspA mediated complement inhibition (Ren et al., 2004a). Furthermore, analysis of the fragments derived from C3 degradation on the pneumococcal surface (C3b and iC3b) showed increased levels of iC3b on the mutant strain, suggesting that PspA prevents processing of C3b into iC3b (Ren et al., 2004a). Finally, it has been shown that PspA-induced inhibition on complement deposition reduces bacterial clearance by phagocytes *in vitro* (Ren et al., 2012).

An additional effect of PspA’s inhibition of complement deposition is the ability to reduce the immune adherence of the bacterium to erythrocytes (as described for the capsular polysaccharides), thus limiting pneumococcal clearance through phagocytosis (Li et al., 2007). This effect was further enhanced in mutant strains lacking PspA and PspC (another complement-interacting protein that will be discussed in the following section), suggesting that these proteins can act synergistically, promoting a strong complement inhibition (Li et al., 2007).

The anti-complement properties of PspA can be abrogated by host immune response against this protein (Ren et al., 2012). A strong correlation between increased survival and the levels of complement deposited onto pneumococci coated with anti-PspA antibodies, has been reported in mice immunized with PspA fragments, suggesting that the blockage of PspA contributes to pneumococcal clearance mediated by complement (Darrieux et al., 2007; Moreno et al., 2010; Goulart et al., 2011). A similar result was obtained using sera from humans immunized with PspA (Ochs et al., 2008). Furthermore, antibodies against PspA purified from mice which had been immunized with heat-killed pneumococci promoted an increase in complement deposition on the bacterial surface (Khan et al., 2015).

Once the bacteria are coated with anti-PspA antibodies and complement, they can be recognized more efficiently by phagocytes and eliminated. Goulart et al. (2011) demonstrated that antibodies induced by mouse immunization with the N-terminal region of PspA from different clades were able to increase phagocytosis of diverse pneumococcal strains by mouse peritoneal phagocytes in presence of a complement source. This effect was confirmed in later studies using different opsonophagocytosis assay (OPA) protocols (Ren et al., 2012; Genschmer et al., 2013) and reinforces the ability of anti-PspA antibodies to promote bacterial phagocytosis through complement’ activation.

#### Pneumococcal Surface Protein C (PspC)

Pneumococcal surface protein C is a multifunctional protein, able to interact with complement components C3 (Cheng et al., 2000), human factor H (FH) (Janulczyk et al., 2000; Dave et al., 2001) and the regulator C4BP (Dieudonne-Vatran et al., 2009), and also functions as an adhesion protein, recognizing secretory immunoglobulin A (SIgA) through the secretory component (SC) (Dave et al., 2004; Kerr et al., 2006). PspC also interacts with a laminin-integrin receptor, improving bacterial adherence and spread to other sites (Orihuela et al., 2009). Moreover, PspC displayed affinity to other components of the extracellular matrix, such as thrombospondin-1 and vitronectin, further enhancing bacterial dissemination (Voss et al., 2013; Binsker et al., 2015).

Pneumococcal surface protein C contains an N-terminal α-helical domain which is exposed at the surface of the bacterium, followed by a proline-rich region and a cell surface-anchoring region (Brooks-Walter et al., 1999). PspCs are classified into 11 groups according to its genetic variation (Iannelli et al., 2002). Different studies demonstrated that PspC from multiple clinical isolates of pneumococci exhibit strong binding to FH (Lu et al., 2006; Moreno et al., 2012).

Pneumococcal surface protein C is able to interact directly with C3 through non-covalent binding to the α and β chains of the C3 molecule, preferentially to the alpha fragment (Cheng et al., 2000). Moreover, it has been shown that PspC interacts with C3 produced by lung epithelial cells, and that this interaction potentiates bacterial adherence (Smith and Hostetter, 2000). The impact of this interaction in pneumococcal disease was confirmed by Kerr et al. (2006) working with infected C3^-/-^ mice; they found that pneumococci lacking PspC presented poor virulence in WT mice, while their potential to cause disease was fully restored in C3 deficient knockout mice.

Pneumococcal surface protein C bound to vitronectin inhibited MAC formation (Jarva et al., 2002; Zipfel et al., 2008; Voss et al., 2013; Kohler et al., 2015), thus acting as a complement regulator, while improving the uptake of pneumococci by host cells (Bergmann et al., 2009). Despite the reduced susceptibility of pneumococci to MAC-mediated lysis, the combined effects of PspC interaction with vitronectin over the complement system contribute to pneumococcal virulence.

Pneumococcal surface protein C is able to recruit FH to the pneumococcal surface with a consequent reduction in complement activation, as demonstrated by an *in vitro* opsonophagocytosis assay (Jarva et al., 2002). PspC-FH interaction has also been shown to occur *in vivo* (Quin et al., 2005). The interaction between PspC and FH inhibits C3bBb formation by competition with factor B (FB) (a Bb precursor) for binding to C3b; it also accelerates irreversible C3bBb decline by relocating Bb, and acts as a cofactor for factor I (FI)-catalyzed C3b cleavage, yielding iC3b that cannot bind FB (Herbert et al., 2015). More recently, the implications of FH-PspC binding were investigated *in vivo*, during experimental human colonization with *S. pneumoniae* (Glennie et al., 2016). The authors found that individuals with epithelial inflammation and increased FH levels (due to influenza virus infection) had increased bacterial loads after experimental colonization, when compared to healthy subjects. Interestingly, the presence of antibodies against PspC did not affect *in vivo* binding of PspC with factor H, possibly because interaction with factor H limits the recognition of PspC by specific antibodies (Glennie et al., 2016). Furthermore, FH is exploited by PspC as a bridge to interact with host cells. Simultaneous binding of FH to PspC on the bacterial surface and the CR3 integrin on polymorphonuclear leukocytes (PMN) and CR3 expressing epithelial cells promoted bacterial adhesion and uptake (Agarwal et al., 2010). While the interaction with PMN may favor bacterial clearance, the increased adherence promoted by PspC interaction with FH is a necessary step for successful colonization (Quin et al., 2007; Agarwal et al., 2010). In fact, the adherence mediated by FH was related to an increased virulence during lung infection in mice (Quin et al., 2007).

In the biofilm phenotype of pneumococci, the expression of PspC was increased as well as the recruitment of FH. Also, the alternative complement pathway activation was impaired, providing a less propitious condition for phagocytosis (Domenech et al., 2013).

The ability of PspC to avoid complement attack was confirmed by Herbert et al. (2015), who found that FH bound to PspC displays a conformational change which causes an enhanced exposure of C3 binding sites (Herbert et al., 2015). As a result, the interaction of PspC-bound FH with C3b was increased twofold, while the alternative pathway convertase disassociation was increased fivefold (Herbert et al., 2015). In summary, PspC’s ability to interact with the complement system is the most important contribution of this molecule to pneumococcal pathogenicity, and provides an interesting target for protein-based vaccines.

#### Pneumococcal Histidine Triad (Pht)

Pneumococcal histidine triad (Phts) is a family of proteins exposed on the surface of *S. pneumoniae.* This group is composed by four members, PhtA, PhtB, PhtD, and PhtE, which are characterized by the presence of histidine triads (HxxHxH) repeated 5 or 6 times in their amino acid sequences (Adamou et al., 2001). Due to the large number of histidine residues in the triads, it has been suggested that Phts may bind to nucleoside or metals. In fact, Phts demonstrate high affinity for divalent cations, with 5 to 6 zinc binding domains (Adamou et al., 2001; Rioux et al., 2011). Zinc is involved in the positive regulation of Phts. Furthermore, variations in zinc concentration on different host niches correlate with the level of Phts expression; Phts are upregulated in the nasopharynx and lungs – where zinc availability is higher – with a reduced expression in blood (Ogunniyi et al., 2009). Despite the variations in Pht levels in different host niches, immunization with Pht fragments is protective in models of bacterial colonization and sepsis (Godfroid et al., 2011).

It has been suggested that Phts interact with complement factor H. However, studies evaluating the binding of Phts to FH *in vitro* revealed conflicting results. Ogunniyi et al. (2009), using FH-enzyme-linked immunosorbent assay and western blot, showed that all Phts bind to factor H, although this interaction was much lower when compared to PspC (positive control). More recently, Melin et al. (2010) used FH-ELISA and demonstrated that Pht proteins did not bind to factor H in most of the serotypes tested; they concluded that the capsule type influences Phts’ ability to interact with FH. In the same work, Melin et al. (2010) used western blot analysis to show that FH was able to bind to cell lysates of Pht^-^ pneumococcal strains, an effect that was dependent on the presence of PspC. The contribution of PspC for FH binding was attested by the abolished binding of this complement regulator, to mutants lacking PspC; in another experiment using flow cytometry, they showed that among five Pht^-^ mutants of different serotypes, only the type 4 strain was able to interact with factor H. They concluded that the relative contribution of Pht proteins to complement inhibition is likely to be affected by the presence of other pneumococcal proteins and, most importantly, depends on the genetic background (Melin et al., 2010).

In summary, the putative role of Phts in the binding to FH remains unclear, although their influence in complement-mediated phagocytosis was in fact observed. Studies with mutant strains negative for all Phts show an increased susceptibility to bacterial opsonization by C3. It was also suggested that a PhtA fragment could cleave human C3 (Hostetter, 1999). Therefore, further studies are needed in order to elucidate the role of Phts in complement-mediated immunity, which should contribute to the evaluation of their potential as pneumococcal vaccines.

#### Murein Hydrolases

Murein hydrolases are enzymes that degrade and remodel the cell wall, and are classified based on the substrate they target. This family of proteins includes amidases, glucosaminidases, muramidases (also termed as lysozymes), endopeptidases and transglycosylases. *S. pneumoniae* exhibits three main murein hydrolases: LytA, LytB, and LytC.

LytA, B, and C are choline binding proteins attached to the pneumococcal surface, involved in pneumococcal cell division – particularly LytB (Garcia et al., 1999) and cell wall remodeling (Gosink et al., 2000; Bai et al., 2014). They have also been shown to improve the adhesion/colonization of human and rat nasopharyngeal cells (Gosink et al., 2000; Bai et al., 2014). The loss of LytB and LytC expression results in an increased susceptibility to phagocytosis by alveolar macrophages and neutrophils (Ramos-Sevillano et al., 2011).

LytA interacts with the complement system in many ways. It binds to C4BP and FH, preventing complement deposition by classical and alternative pathways (Ramos-Sevillano et al., 2015). It has also been demonstrated that LytA inhibits binding of C1q to CRP, which was related with an increased exposure of Phosphocholine molecules in the LytA-negative mutant strain (Ramos-Sevillano et al., 2015). LytA can cleave C3b and iC3b fragments deposited in the bacterial surface regardless of the presence of capsule (Ramos-Sevillano et al., 2015), reducing opsonic activity and subsequent phagocytosis. Indeed, phagocytosis by neutrophils and alveolar macrophage cells was described to be higher in the *lytA* mutant strain (Ramos-Sevillano et al., 2012). The mutant strain also displayed an increased complement deposition when compared with the parental strain expressing autolysin (Ramos-Sevillano et al., 2012).

LytC negative pneumococci show an increased C3b deposition on their surface, an effect that was not observed with the LytB mutant (Ramos-Sevillano et al., 2011). However, despite the absence of a direct effect of LytB alone on complement deposition, a double mutant lacking LytB and LytC revealed a higher sensitivity to C3b deposition when compared with the single LytC mutant (Ramos-Sevillano et al., 2011). This suggests a contribution of both proteins to control complement deposition on pneumococci, an effect that was related to increased virulence in models of colonization and invasive disease in presence of LytB and LytC (Ramos-Sevillano et al., 2011).

Despite the evidence of LytA, LytB and LytC involvement in complement resistance by *S. pneumoniae*, the exact mechanism by which they confer resistance to complement is not completely clear. It is possible that the chain-dispersing function of these hydrolases influences the amount of C3 deposition on the bacterial surface. Growing of *S. pneumoniae* in biofilms was shown to reduce complement deposition and confer resistance to its opsonic activity and subsequent phagocytosis, when compared to bacteria in diplococci morphology. Indeed, the absence of LytA and LytB was found to promote formation of long chains of pneumococci, while the WT strain yielded diplococci morphology (Domenech et al., 2013). In addition, treatment of long pneumococcal chains with LytB led to bacterial dispersion into diplococci or short chains (De Las Rivas et al., 2002; Dalia and Weiser, 2011; Domenech et al., 2013). This result suggests a link between colony morphology and complement deposition on pneumococci, while indicating a role for autolysins in this mechanism.

#### Pneumolysin (PLY)

Pneumolysin (PLY) is a cytotoxic protein produced by all strains of *S. pneumoniae*, found in the cytosol and cell wall compartments (Price and Camilli, 2009), and also being released from the bacterium during cell growth (Balachandran et al., 2001). PLY displays many important functions during pneumococcal infections, including (i) hemolytic and genotoxic effects (Johnson et al., 1980; Rai et al., 2016); (ii) induction of apoptosis in different cells – including macrophages and brain cells (Braun et al., 2001; Srivastava et al., 2005); (iii) interaction with TLR-4 and production of inflamatory cytokynes (Malley et al., 2003); (iv) activation of NLRP3 inflamasome (McNeela et al., 2010); (v) induction of neutrophil extracelullar trap (NET) formation (Nel et al., 2016); (vi) inhibition of cilliary movements in epithelial respiratory cells (Rayner et al., 1995; Marriott et al., 2008) and (vii) complement activation (Paton et al., 1984; Mitchell et al., 1991; Benton et al., 1997; Alcantara et al., 2001; Jounblat et al., 2003; Yuste et al., 2005; Propst-Graham et al., 2007; Marriott et al., 2008; Ali et al., 2013).

Pneumolysin’s ability to activate complement – which could be interpreted as disadvantageous for pneumococci – has actually been reported as a protective mechanism for the pathogen; since PLY is only exposed to interact with complement when it is released from the bacterium (the cell wall-attached molecule is not accessible to recognition by antibodies or complement), it has been suggested that complement activation by PLY causes depletion of complement components in the serum (Paton et al., 1984; Mitchell et al., 1991), thus limiting complement binding to the pneumococcus. This effect was confirmed *in vivo* using mutant strains that produce truncated forms of the toxin (Rubins et al., 1995; Alcantara et al., 2001; Propst-Graham et al., 2007); in a lung infection model, a mutant strain expressing a PLY which lacks the complement activation activity displayed a marked reduction in bacterial loads in the mice lungs (Rubins et al., 1995; Propst-Graham et al., 2007). Similarly, complementation of a PLY negative mutant with a recombinant PLY presenting intact complement activation capacity but no hemolytic activity was able to restore the bacterial virulence, indicating an independent contribution of the complement activation motif of PLY for pneumococcal virulence in this infection model (Rubins et al., 1995). However, in models of pneumococcal bacteremia, a significant role for the complement activity of PLY was only observed in cirrhotic rats (which produce reduced amounts of complement), with no effect in animals exhibiting normal complement production (Alcantara et al., 1999). This result suggests that the contribution of the complement activation motif of PLY is important in host niches or systems where a limited amount of complement is available (Propst-Graham et al., 2007; Marriott et al., 2008).

Pneumolysin’s ability to activate complement occurs mainly through the classical pathway; in fact, a direct interaction of PLY with C1 has been described *in vitro* (Paton et al., 1984) and related to a specific region of PLY that shows homology to human CRP, located in the domain 4 (Mitchell et al., 1991). PLY also shows structural similarity with the Fc portion of immunoglobulin, being able to bind Ig (Mitchell et al., 1991). Interestingly, the region involved in interaction with Ig is the same responsible for PLY self-oligomerization, suggesting one mechanism by which PLY promotes the complement activation [reviwed in (Marriott et al., 2008)]. However, complement activation by PLY is not dependent on the presence of specific anti-PLY antibodies, since sera from unvaccinated humans and non-immune mice showed increased C3 deposition on pneumococci lacking PLY (Yuste et al., 2005). Additionally, the contribution of PLY to complement-mediated inflammatory response may be enhanced by the fragments generated from complement activation (C3a and C5a), which attract polymorphonuclear cells, as already seen in serum containing high amounts of PLY (Johnson et al., 1981).

The observation that human serum (but not mice) depleted from C1q still promotes PLY opsonization by C3 activation products suggests a role for the lectin pathway in PLY-induced complement activation (Ali et al., 2013). In fact, human L-ficolin has been shown to initiate MBL activation by PLY in human serum, contributing to the classical pathway activation (Ali et al., 2013).

In addition to its direct role in complement activation, PLY participates in the assembly of pneumococcal biofilms (Shak et al., 2013) – a phenotype that is important for successful colonization and during the early stages of invasive infections, as well as limiting the interaction of complement components with surface molecules of pneumococci (Hall-Stoodley et al., 2006; Domenech et al., 2013). Therefore, the involvement of PLY on biofilm formation indirectly contributes to evasion from complement.

In summary, the contribution of PLY to pneumococcal virulence – which is highly influenced by the ability to interact with the complement system – reinforces the importance of complement evasion for bacterial survival, as well as PLY’s potential as a vaccine candidate against this pathogen.

#### Pneumococcal Exoglycosidases

The pneumococcal exoglycosidases are a group of enzymes that cleave terminal sialic acid from glycoconjugates. Among five exoglycosidases expressed by pneumococci – NanA, NanB, NanC, BgaA, and StrH, three surface associated molecules have been shown to interact with complement: NanA, BgaA, and StrH.

Neuraminidase A (NanA) is able to remove the sialic acid (SA) from a range of host substrates, thereby promoting the interaction between pneumococci and host cell receptors that contribute to pneumococcal colonization (Tong et al., 2000; Brittan et al., 2012). BgaA is the only pneumococcal β-galactosidase; it is secreted and acquired by the pneumococcal surface through a sortase. BgaA exhibits affinity to terminal galactose β (1–4) linked to GlcNAc, suggesting a role in deglycosylation of glycoconjugates of human cells (?) and was shown to be an adhesin, regardless of its galactosidase activity (Limoli et al., 2011). *N*-acetylglucosaminidase (StrH) is an exoglycosidase found in the bacterial surface and supernatant, able to cleave *N*-acetylglucosamine (GlcNAc) that is linked to mannose through β-1 (Zähner and Hakenbeck, 2000). All three proteins have been reported to work sequentially in the deglycosylation of SA, galactose, *N*-acetylglucosamine, decrypting mannose of host glycoproteins, which favors adherence/colonization (King et al., 2006).

These proteins have also been shown to deglycosylate human glycoconjugates as lactoferrin and IgA (King et al., 2006), demonstrating a direct interaction with the immune system. Indeed, SA participates in several immune processes such as leukocyte rolling, cell activation, modulation of immune cell functions and interactions with the complement system [reviewed in (Varki and Gagneux, 2012)].

NanA, BgaA, and StrH were shown to act in conjunction to limit complement deposition on pneumococci of different genetic backgrounds (Dalia et al., 2010). Blocking of the classical pathway did not affect complement levels on the bacterial surface, suggesting that NanA acts on the alternative pathway (Dalia et al., 2010). These proteins were also shown to inhibit bacterial killing by human phagocytes *in vitro*. This effect was attributed to the sequential removal of sugar moieties from host glycoconjugates by NanA, BgaA, and StrH: NanA removes sialic acid that is α2–3 or α2–6 linked to galactose, BgaA excises the galactose that is β1–4 linked to *N*-acetylglucosamine, and StrH removes *N*-acetylglucosamine that is β1 linked to mannose (King et al., 2006). Since complement components contain many glycosylation sites which are necessary for their functions [reviewed in (Ritchie et al., 2002)], the sequential activities of NanA, BgaA, and StrH possibly deglycosylate one or more glycoproteins that are important for complement deposition, thus leading to inhibition of complement mediated opsonophagocytosis (Dalia et al., 2010).

Previous studies found a role for SA in subversion of complement alternative pathway. SA is required for effective binding between FH and surface-attached C3b, as well as for dissociation of C3 convertase in sheep erythrocytes (Fearon, 1978). Pathogens like *Neisseria meningitidis* and *Haemophilus influenzae* exploit this complement-evasion strategy by producing SA that binds factor H (Ram et al., 1998). Interestingly, NanA from *S. pneumoniae* is able to remove sialic acid from the surface of these pathogens, thus limiting their ability to escape complement attack, providing a competitive advantage for pneumococci (Shakhnovich et al., 2002).

In summary, despite the evidence of exoglycosidases role in complement evasion, further investigations are required in order to elucidate the exact mechanism by which these proteins circumvent complement activation.

#### Pneumococcal Pilus Adhesine (RrgA)

*Streptococcus pneumoniae* expresses a pilus-like structure, encoded by the rlrA pilus islet 1, which contributes to virulence in animal models of pneumococcal infection (Barocchi et al., 2006). This structure is composed of three proteins: RrgA, RrgB, and RrgC (Barocchi et al., 2006; Hilleringmann et al., 2009). RrgA is an adhesin that interacts with lung epithelial cells *in vitro* and confers virulence in mice (Barocchi et al., 2006; Hilleringmann et al., 2009). It is also able to increase bacterial phagocytosis by murine and human cells (Hilleringmann et al., 2009). Orrskog et al. (2012), using flow cytometry have shown that purified complement receptor 3 (CR3) binds to pneumococci expressing RrgA, and that purified RrgA is able to interact with the CD11b integrin domain of CR3. In addition, RrgA expression was linked to a faster dissemination of pneumococci from the upper respiratory tract and peritoneal cavity to the bloodstream of challenged mice, and to an increased bacterial survival inside macrophages (Orrskog et al., 2012). Altogether, the evidence indicate that RrgA interaction with CR3 on the surface of macrophages promotes bacterial internalization, while a prolonged survival of the internalized pneumococci contributes to their dissemination during systemic infection.

#### Pneumococcal Moonlighting Proteins

Moonlighting proteins are a group of molecules able to perform distinct biological activities through a single domain. Found in both prokaryotic and eukaryotic cells, these proteins display several activities, including roles in metabolic processes, host-pathogen signaling, transcription activation, adhesion and invasion of host tissues (Henderson and Martin, 2014). Based on their potential to interact with host cells, bacterial moonlighting proteins are important virulence factors in prokaryotic organisms (Jeffery, 1999; Henderson and Martin, 2011).

One important feature shared by many moonlighting proteins is their ability to interact with plasminogen (PLG) (Henderson and Martin, 2011), a plasmatic glycoprotein that generates plasmin (PL) upon activation. PL is a serine protease which displays many functions in tissue remodeling and coagulation. The conversion of PLG into PL is accomplished by activators like urokinase type A (uPA) and tissue type plasminogen (tPA) activators (human molecules) or by bacterial proteins [reviewed in (Lahteenmaki et al., 2001)].

Plasminogen and PL interfere with many molecules of the complement system (Barthel et al., 2012; Castiblanco-Valencia et al., 2016). PL cleaves C1, C2, C3, C3b, C4, and C5 (Pillemer et al., 1953; Amara et al., 2010; Barthel et al., 2012), thereby depleting and inhibiting complement activation through all three complement pathways. Even the enzymatically inactive PLG was able to improve the cofactor activity of FH in the inactivation of C3b by Factor I (Foley et al., 2015). It also cleaves iC3b, yielding C3dg-like, which acts as an opsonin and an activator of proinflammatory cytokines in macrophages (Foley et al., 2015). PLG has been found to interact with C3, C5, C3b, C3c, and C3d (Barthel et al., 2012).

*Streptococcus pneumoniae* expresses moonlighting proteins (Henderson and Martin, 2013), including PepO, enolase, GAPDH, PGK, and Tuf, which are able to acquire surrounding PLG molecules of the host. This interaction has been shown to contribute to invasion of host tissues and to avoid complement attack. The actions of pneumococcal moonlighting proteins are described in more detail below.

##### Endopeptidase O (PepO)

Endopeptidase O (PepO) displays a HEXXH motif, an important active site of neutral endopeptidase (NEP) and typical of zinc-dependent metallopeptidases (Rawlings and Barrett, 1995). NEP is a member of M13 peptidase family, involved in many physiological functions and immune responses. Found on the pneumococcal surface and in culture supernatants, endopeptidase O has been reported to bind plasminogen and fibronectin (constituent of human extracellular matrix) (Agarwal et al., 2013b), thereby coating the pneumococcal surface with these molecules, which facilitate pneumococcal adhesion and invasion of host cells, while inhibiting complement-mediated immunity. Indeed, the binding of PepO to PLG in the presence of uPA was able to cleave C3b (Agarwal et al., 2013b). In addition, PepO has been found to bind to both C1q (C-terminal globular heads) and C4BP (CCP8 of the α-chain) through ionic interactions (Agarwal et al., 2014), with direct implications on complement activation. The ability to bind C1q upon release of PepO promotes complement activation and rapid consumption of complement components around the bacterium, with a consequent inhibition of complement activation on the pneumococcal surface (Agarwal et al., 2014). Further inhibition of complement activation is achieved by interaction with the complement regulator C4BP (Agarwal et al., 2014). Also, the interaction of surface exposed PepO with C1q increases pneumococcal adherence to host epithelial cells *in vitro* (Agarwal et al., 2013a, 2014; Agarwal and Blom, 2015).

##### α-Enolase (Eno)

Pneumococcal α-Enolase (Eno) is present in virtually all strains, found in the bacterial cytoplasm, displayed on the surface and released to the culture supernatant. The mechanism of Eno exportation is not completely elucidated, although it does not seem to be dependent on autolysis (Bergmann et al., 2003). It is a glycolytic enzyme involved in conversion of 2-phospho-D-glycerate to phosphoenolpyruvate during glycolysis (Whiting et al., 2002). Similar to other moonlighting proteins, it has been shown to play a role in bacterial adherence to host tissues and invasion by binding to PLG and, consequently, promoting degradation of extracellular matrix components (Bergmann et al., 2005; Bergmann et al., 2013). Eno-deficient pneumococcal mutants displayed reduced binding to PLG, which correlated with a reduced adherence to host cells *in vitro* (Bergmann et al., 2013).

Pneumococcal Eno exhibits additional roles in virulence. It was reported to ionically bind to C4BP (Agarwal et al., 2012), consequently coating the surface of pneumococci with this complement regulatory molecule. The binding sites were identified in the CCP domains of α-chain in C4BP. It was also reported that Eno can concomitantly bind to C4BP and PLG in a non-competitive fashion, increasing the activation of PLG into plasmin (Agarwal et al., 2015). Furthermore, C4BP bound to Eno on the pneumococcal surface acted as a cofactor in C4d degradation by FI, while leading to a reduction in C3b deposition on the bacterium (Agarwal et al., 2012). Additionally, Eno has been shown to interact with neutrophils promoting formation of neutrophil extracellular traps (NETs) which increased their bactericidal effect (Mori et al., 2012). One possible explanation for this apparently disadvantageous interaction is that NET-induced host tissue damage may favor the spread of the microorganism; however, this effect has not been investigated to date.

##### Glyceraldehyde-3-phosphate Dehydrogenase (GAPDH)

Glyceraldehyde-3-phosphate Dehydrogenase is a glycolytic enzyme shared by prokaryotic and eukaryotic cells which has been implicated in the pathogenesis of bacterial infections (Jin et al., 2011), acting as an adhesion (Pancholi and Fischetti, 1992; Zhang et al., 2015), a transferrin binding molecule (in both bacteria and human cells) (Modun and Williams, 1999; Raje et al., 2007; Kumar et al., 2012) as well as in host immune modulation and apoptosis (Terao et al., 2006; Terrasse et al., 2012).

Although first identified as a cytoplasmic protein, it has been demonstrated that pneumococcal GAPDH is surface-associated and surface-exposed (Terrasse et al., 2012, 2015), where it can act as an adhesin and bind PLG (Attali et al., 2008). Binding of PLG to pneumococci increased bacterial adherence and invasion of host cells (Attali et al., 2008). GAPDH released through autolysis is also able to bind C1q (Terrasse et al., 2012) and to activate complement. Pneumococci lacking the glycolytic enzyme exhibited reduced binding to PLG and deposition of C1q, C3 and C4 components (Terrasse et al., 2012), evidencing a role of GAPDH in the activation and subsequent deposition of complement.

Glyceraldehyde-3-phosphate Dehydrogenase was reported to bind directly to peptidoglycan regardless of the presence of teichoic or lipoteichoic acids, though a mechanism dependent on LytA-mediated lysis (Terrasse et al., 2015). In the same study, peptidoglycan bound GAPDH showed an increased C1q deposition (Terrasse et al., 2015); however, C1q binding in the presence of GAPDH was not able to activate the complement cascade, suggesting that other molecules may be involved in complement activation in this model (Terrasse et al., 2015).

The implications of binding of GAPDH (free form) to C1q may be similar to those found for PepO, leading to depletion of complement components away from the bacterium, and consequently avoiding complement mediated pneumococcal clearance. However, the precise mechanism of GAPDH – attached to the pneumococcal surface or not – interaction with the complement system has not been investigated.

##### Phosphoglycerate Kinase (PGK)

Phosphoglycerate Kinase is a glycolytic enzyme involved in the conversion of 1,3-biphosphoglycerate to 3-phosphoglycerate (Bernstein et al., 1998). As reported for other moonlighting proteins, PGK can be found in supernatants, although the precise mechanism of secretion and surface exposure of this molecule remains unclear. Another similarity with the moonlighting proteins is the ability to bind PLG. Interestingly, in addition to PLG, PGK interacts simultaneously with tissue plasminogen activator – tPA (Fulde et al., 2014), which is responsible for PLG conversion into plasmin, leading to degradation of extracellular matrix proteins and contributing to bacterial invasion and dissemination, while hindering the opsonic effect of the complement system (Fulde et al., 2014).

Furthermore, PKG has recently been reported to bind ionically and simultaneously to MAC proteins (Blom et al., 2014). Pneumococcal MAC-mediated killing, as occurs with other Gram-positive microbes, is found to be ineffective, due to an increased resistance to insertion of C5b9 in the membrane protected by a thick peptidoglycan (Blom et al., 2014). The components of the terminal pathway C5, C7, and C9 were found to interact with PKG, undermining MAC assembly as a result of complement components depletion. Indeed, PGK associated with the pneumococcal surface promoted an inhibition of MAC deposition. These interactions were also able to reduce the hemolytic capacity of human serum against the bacterium. Moreover, PLG bound to PGK was activated in PL, which cleaved C3b (Blom et al., 2014). The impaired assembly of MAC takes place during the initial (by binding C5 and C7) and late phases (by binding C9) of the terminal pathway. The binding domains of C5 and C7 appear to be partially superimposed, while the C9 site in PGK is not; this latter interaction is capable of inhibiting the C9 self-polymerization (Blom et al., 2014).

In summary, the many sites of interaction between PGK and complement proteins suggest a role in pneumococcal ability to avoid complement attack, since complement proteins interacting with PGK ultimately become unavailable to participate in the cascade.

##### Elongating factor Tu (Tuf_sp_)

The elongating factor Tuf_sp_ is a surface-accessible protein from *S. pneumoniae*, also found in the bacterial cytoplasm and culture supernatants. It is a conserved protein found in different species of bacteria and fungi, including pathogenic and non-pathogenic organisms (Jacobson and Rosenbusch, 1976; Crowe et al., 2003; Archambaud et al., 2005; Gross and Kinzy, 2005; Jonak, 2007; Li et al., 2013; Mohan et al., 2014). Tuf exhibits molecular chaperone activity, being involved in peptide biosynthesis, protein folding and cell response to stress, promoting protein renaturation (Gregersen and Bross, 2010). It is also involved in the transport of amino-acyl-tRNA to ribosomes (Murase et al., 2003; Nilsson and Nissen, 2005).

Pneumococcal Tuf was described by Mohan and co-workers as a factor H-binding protein, identified through mass spectrometry of a gel band from a mutant pneumococcus lacking PspC, which still reacted with Factor H by western blot (Mohan et al., 2014). The domains of FH responsible for the interaction were CCP 6–7 and 18–20 (heparin site). Tuf was also able to bind to other complement regulators, FHL-1 (a short form of FH) and to Factor H related protein 1 (CFHR-1) (CCP 3–5). The complement regulatory activity promoted by FH and FHL-1 as cofactors of FI was maintained when they were bound to Tuf. Moreover, the concomitant binding of Tuf and FH to plasminogen promotes plasmin formation in presence of uPA, which in turn cleaves fibrinogen, C3 and C3b (Mohan et al., 2014). The combined effects of complement inhibition and degradation of ECM components promoted by interaction of Tuf with its many ligands enhance the bacterium’s ability to invade host tissues.

## Complement-Interacting Pneumococcal Proteins as Vaccines Candidates

The efficacy of the polysaccharide-based vaccines is attributed to their ability to induce serotype-specific antibodies that promote bacterial phagocytosis, a mechanism that is largely dependent on complement activation (Song et al., 2013). This result reinforces the importance of the complement system to vaccine-induced protection against pneumococcal infection.

Several pneumococcal proteins have been investigated as potential candidates for inclusion in a future pneumococcal vaccine, with encouraging results (Hamel et al., 2004; Gor et al., 2005; Gianfaldoni et al., 2007; Ferreira et al., 2009; Godfroid et al., 2011; Hernani Mde et al., 2011; Kamtchoua et al., 2013; Darrieux et al., 2015). PspA, PspC, Phts, NanA, PLY, RrgA, LytA, and GAPDH have all been demonstrated to induce protection against systemic infection [reviewed in (Darrieux et al., 2015) and (Miyaji et al., 2013)]; PspC, Phts, NanA, and PspA were also protective against nasopharyngeal colonization (Arulanandam et al., 2001; Balachandran et al., 2002; Tong et al., 2005; Hernani Mde et al., 2011), while NanA protects chinchillas against otitis media (Long et al., 2004).

Moreover, the role of complement activation as a protective mechanism promoted by vaccination has been exploited in many studies, including animal models of colonization and invasive disease (Darrieux et al., 2007, 2015; Ogunniyi et al., 2007; Ferreira et al., 2010; Moreno et al., 2010; Paton, 2011; Miyaji et al., 2013). The surface association or microbial release of these proteins further supports their potential as vaccine candidates, allowing immune recognition in vaccinated individuals upon contact with the pathogen. Finally, PspA, PspC, Phts, and Pds (derived from genetically or chemically detoxified PLY) have also been evaluated in clinical trials, and shown to be immunogenic and safe [reviewed in (Darrieux et al., 2015)].

In order to improve vaccine immunogenicity and coverage, combinations of these antigens have been used, with promising outcomes. PspA and PspC have been shown to confer broad protection against colonization and invasive diseases (Ferreira et al., 2009). The inclusion of Phts also promotes potent and protective immune responses. PspA and Pds were able to augment protection against lethal pneumococcal sepsis (Briles et al., 2000). Furthermore, the genetic fusion of pneumococcal antigens has been employed as an alternative strategy to broaden vaccine coverage, while reducing steps in the production process. This is particularly important when considering structurally variable antigens – such as PspA (which is the most promising vaccine candidate so far, but exhibits serological diversity which limits vaccine coverage). In the case of PspA, genetic fusion of molecules from different families (Darrieux et al., 2007), or fusion with Pds (Goulart et al., 2013), showed an enhancement in vaccine coverage.

## Conclusion

The high numbers of pneumococcal proteins which are capable of interacting with the complement system highlight the importance of this defense mechanism to limit the diseases caused by this pathogen. Many of these proteins act on the same step of the complement cascade; this redundancy suggests that the loss of one antigen may be compensated by another protein with similar functions.

Several pneumococcal proteins have been evaluated as potential vaccine candidates, with variable outcomes. The emerging concept regarding protein-based pneumococcal vaccines (which has been expanded to other pathogenic bacteria and protozoa) is that the use of one protein alone may not be sufficient to provide long term, wide protection against pneumococcal diseases [reviewed in (Darrieux et al., 2015)]. Therefore, the inclusion of different antigens in a multi-component vaccine formulation has been emphasized as a promising approach to prevent pneumococcal diseases. In this sense, the combination of antigens that act over the same mechanism may be an effective strategy to block immune evasion by the pathogen, allowing for rapid bacterial clearance. Therefore, we suggest that the combination of pneumococcal proteins that act by limiting complement activation on *S. pneumoniae* could provide strong protection against infections caused by this pathogen. In particular, different combinations of PspA, PspC, Phts e PLY (up to three proteins in one formulation) have already been successfully evaluated in animal models and clinical trials. However, the inclusion of high numbers of antigens in a single formulation raises concerns regarding protein interference, and also imposes production limitations. An alternative would be the use of protein chimeras – including immunogenic fragments of the virulence factors genetically fused – which have been shown to preserve individual antigenic properties and even expose previously cryptic epitopes, while increasing vaccine coverage. This would allow the inclusion of new vaccine candidates – such as the moonlighting proteins – which can further enhance vaccine efficacy. Nevertheless, more studies are needed in order to determine the protective potential of this vaccine approach.

## Author Contributions

GA, TC, WP, and MD drafted the manuscript. LF, MR, LL, and MD reviewed the text. All authors read and approved the final manuscript.

## Conflict of Interest Statement

The authors declare that the research was conducted in the absence of any commercial or financial relationships that could be construed as a potential conflict of interest.

The handling Editor declared a shared affiliation, though no other collaboration, with the authors LL, TC and states that the process nevertheless met the standards of a fair and objective review.
