# Supplementary material for: Role of Streptococcus pneumoniae Proteins in Evasion of Complement-Mediated Immunity
Source: Front Microbiol. 2017 Feb 20;8:224. doi: 10.3389/fmicb.2017.00224 (PMC5316553; doi:10.3389/fmicb.2017.00224)
Supplement: Supplementary file 1 [file Table_1.DOCX]

| **Table 1. Proteins of complement cascade.** | | | | | | | | | |
| --- | --- | --- | --- | --- | --- | --- | --- | --- | --- |
|  | | |  | | **Classical pathway** | | | | |
| **Components** | | | | | **Functions** | | | | |
| C1 | | | Complex composed by C1q, C1r (2), C1s(2) | | Recognition molecule and initiator of complement cascade. | | | |  |
| C1q | | | Subunit of C1 complex | | Binds to Fc region of antibodies from immune complexes. | | | |  |
| C1r | | | Subunit of C1 complex | | Serine protease. Cleaves and activates C1s molecules. | | | |  |
| C1s | | | Subunit of C1 complex | | Serine protease. Cleaves C4 and C2. | | | |  |
| C4 | | | Plasma protein | | Precursor of C4b. | | | |  |
| C4a | | | C4 cleavage product | | Traditionally depicted as an anaphylatoxin, current data refutes this hypotesis*. | | | |  |
| C4b | | | C4 cleavage product | | Attaches covalently to cell surface; acts as binding platform for C2a. | | | |  |
| C2 | | | Plasma protein | | Precursor of C2a. | | | |  |
| C2a | | | C2 cleavage product | | Protease serine with the enzymatic action of C3 convertase. Binds C4b. | | | |  |
| C2b | | | C2 cleavage product | | Free fragment. | | | |  |
| C4bC2a | | | Classical pathway C3 convertase | | Cleaves C3 into C3b and C3a. | | | |  |
| SIGN-R1 | | | Transmembrane type-C lectin | | Recognizes *S. pneumoniae* surface and binds to C1q to initiate the cascade. | | | |  |
|  | | |  | |  |  | | | |
| **Lectin pathway** | | | | | | | | | |
|  | **Components** | | | | **Functions** | | | | |
| MBL | | | | Mannose binding lectin | Structurally similar to the C1q. Recognition molecule and initiator. | | |  | |
| MASP-1 | | | | Mannose-associated serine protease 1 | Self-activates. Cleaves C2 and C3 (less efficient). | | |  | |
| MASP-2 | | | | Mannose-associated serine protease 2 | Self-activates. Cleaves C2 and C4. | | |  | |
| M-Ficolin | | | | or Ficolin-1. A collectin | Similar to MBL, together with MASPs acts as a recognition molecule. | | |  | |
| L-ficolin **^a^** | | | | or Ficolin-2. A collectin | Similar to MBL, together with MASPs acts as a recognition molecule. | | |  | |
| H-Ficolin | | | | or Ficolin-3. A collectin | Similar to MBL, together with MASPs acts as a recognition molecule. | | |  | |
| CL-11/CL-K1 **^a^** | | | | or Collectin-11 or collectin kidney 1 | Similar to MBL, together with MASPs acts as a recognition molecule. | | |  | |
| CL-L1 | | | | or Collectin-10 or collectin liver 1 | Similar to MBL, together with MASPs acts as a recognition molecule. | | |  | |
|  | | | |  |  | |  | | |
|  | | | |  | **Alternative pathway** | | | | |
|  | | **Components** | | | **Functions** | | | | |
| C3 **^b^** | | | | Plasma protein | Precursor of C3b. | | |  | |
| C3(H_2_0) | | | | Hydrolyzed C3 | Initiating molecule of fluid phase C3 convertase. Attaches to cell surfaces. | | |  | |
| C3b | | | | C3 cleavage product | Acts as opsonin alone and participates in C3 and C5 convertase. | | |  | |
| C345C | | | | Domains of C3, C4, C5 with 3 disulfide bonds | Inside C3, this is the target of Properdin, which upregulates the cascade. | | |  | |
| C3a | | | | C3 product cleaved by C3 convertase | Free anaphylatoxin. | | |  | |
| FB | | | | Factor B. Plasma protein. | Precursor of Bb. | | |  | |
| Ba | | | | FB product when cleaved by FD | Free fragment. | | |  | |
| Bb | | | | FB product when cleaved by FD | Serine protease with the enzymatic action of C3 convertase. Binds C3b. | | |  | |
| FD | | | | Factor D | Serine protease. Cleaves FB bound to C3b molecule yielding Ba and Bb. | | |  | |
| C3(H_2_0)Bb | | | | Soluble phase C3 convertase | Complex generated by C3 tickover which cleaves C3 into C3b and C3a. | | |  | |
| C3bBb | | | | Surface attached C3 convertase | Generated by binding of C3b and Bb. Cleaves C3 into C3b and C3a. | | |  | |
| iC3b | | | | C3b product cleaved by FI | Generated by regulation of FI. Acts as opsonin. | | |  | |
| C3d | | | | C3b product cleaved by FI | Generated by regulation of FI. B cell activator. | | |  | |
| P | | | | Properdin | Positive regulator. Attaches to C3b and stabilizes the C3 convertase. | | |  | |
| **Terminal pathway** | | | | | | | | | |
| **Components** | | | | | **Functions** | | | | |
| C4bC2aC3b | | | C5 convertase of classical and lectin pathway | | Protein complex which cleaves C5 molecules yielding C5a and C5b. |  | | | |
| C3bBbC3b | | | C5 convertase of alternative pathway | |  |  | | | |
| C5 | | | Plasma protein | | C5b precursor, participates in MAC assembly. |  | | | |
| C5a | | | C5 product cleaved by C5 convertase | | Free anaphylatoxin. |  | | | |
| C5b | | | C5 product cleaved by C5 convertase | | Initiator molecule of MAC assembly. |  | | | |
| C6 | | | Plasma protein | | Binds to C5b and recruits C7. |  | | | |
| C5b-6 | | | Protein aggregate | | Complex formed by C5b and C6 molecules. |  | | | |
| C7 | | | Plasma protein | | Binds to C5b-6 complex. |  | | | |
| C5b-7 | | | Protein aggregate | | Complex formed by C5bC6 and C7 molecule. |  | | | |
| C8 | | | Plasma protein | | Binds to C5b-7. |  | | | |
| C5b-8 | | | Protein aggregate | | Complex formed by C5bC6C7 and C8 molecule. |  | | | |
| C9 | | | Plasma protein | | Binds to C5b-8. Responsible for pore formation onto cell surfaces. |  | | | |
| C5b-9 | | | Protein aggregate | | Complex formed by C5bC6C7C8 and C9 molecules. |  | | | |

**^a^** Recognizes *S. pneumoniae*.

**^b^** It is common for all the pathways.

* Barnum, S.R. (2015). C4a: An Anaphylatoxin in Name Only. J Innate Immun 7(4), 333-339. doi: 10.1159/000371423.
